# Frequency of occurrence and antimicrobial susceptibility of bacteria isolated from respiratory samples of patients hospitalized with pneumonia in Western Europe, Eastern Europe and the USA: results from the SENTRY Antimicrobial Surveillance Program (2016–19)

**DOI:** 10.1093/jacamr/dlab117

**Published:** 2021-09-02

**Authors:** Helio S Sader, Jennifer M Streit, Cecilia G Carvalhaes, Michael D Huband, Dee Shortridge, Rodrigo E Mendes, Mariana Castanheira

**Affiliations:** JMI Laboratories, North Liberty, IA 52317, USA

## Abstract

**Background:**

The SENTRY Antimicrobial Surveillance Program monitors the frequency of occurrence and antimicrobial susceptibility of organisms from various infection types worldwide.

**Objectives:**

To evaluate the SENTRY programme results for organisms isolated from respiratory samples of patients hospitalized with probable pneumonia.

**Methods:**

A total of 28 918 bacterial isolates were consecutively collected (one per patient) in 2016–19 from 121 medical centres located in western Europe (W-EU; *n* = 7966), eastern Europe (E-EU; *n* = 3182) and the USA (*n* = 17 770) and then susceptibility tested by reference broth microdilution methods in a central laboratory.

**Results:**

Gram-negative bacilli (GNB) represented 76.3%, 88.6% and 69.1% of organisms; non-fermentative (NF) GNB accounted for 26.9%, 51.8% and 34.6% of organisms in W-EU, E-EU and USA, respectively. *Pseudomonas aeruginosa* susceptibility to piperacillin/tazobactam and meropenem was 75.4% and 76.9% in W-EU, 57.4% and 48.3% in E-EU, and 76.1% and 74.8% in the USA, respectively. Only 10.4% of *Acinetobacter baumannii* isolates from E-EU were meropenem susceptible compared with 45.8% in W-EU and 58.8% in the USA. Overall MRSA rates were 21.4% in W-EU and 28.7% in E-EU. In the USA, MRSA rates decreased from 44.8% in 2016 to 40.1% in 2019. Carbapenem resistance among Enterobacterales decreased continuously in the USA from 3.0% in 2016 to 1.7% in 2019 (2.4% overall) and was higher in E-EU (16.6%) than W-EU (2.2%). *Klebsiella pneumoniae* susceptibility to meropenem was 91.3%, 72.5% and 95.3% in W-EU, E-EU and the USA, respectively.

**Conclusions:**

Rank order and antimicrobial susceptibility of bacteria isolated from patients with pneumonia widely varied by geography. MDR NF-GNB represented an important cause of pneumonia.

## Introduction

The SENTRY Antimicrobial Surveillance Program monitors the frequency of predominant pathogens and the antimicrobial resistance patterns of nosocomial and community-onset infections via a broad network of sentinel hospitals distributed worldwide. SENTRY is the longest running antimicrobial surveillance programme that globally monitors pathogens and the changes in resistance patterns over time through centralized testing utilizing reference susceptibility methods. In SENTRY, bacterial isolates are consecutively collected (one per infection episode) according to the infection type and sent to a central monitoring laboratory (JMI Laboratories, North Liberty, IA, USA) where the isolates are tested for susceptibility by reference broth microdilution methods against many antimicrobial agents available for clinical use.^1^

Pneumonia is the second most common infection in hospitalized patients, and it is associated with significant morbidity and mortality.^2^^,^^3^ The National Healthcare Safety Network (NHSN) programme follows the frequency and antimicrobial susceptibility of bacteria causing various types of infection in US medical centres, including healthcare-associated bacterial pneumonia;^4^ however, large surveillance programmes on healthcare-associated pneumonia are scarce in other parts of the world.^5^ The frequency and antimicrobial susceptibility patterns of pathogens collected from patients hospitalized with bacterial pneumonia worldwide during the first 20 years of SENTRY (1997–2016) were published a few years ago.^6^ That investigation clearly showed how rank order and susceptibility rates varied widely by geographic region and over time, as some resistance phenotypes increased while others decreased. Those results emphasized the need for continued surveillance. In the present investigation, we updated the previously published data by evaluating bacterial isolates collected between 2016 and 2019 from medical centres located in the USA and Europe. Moreover, the European countries were divided into two groups, western Europe (W-EU) and eastern Europe (E-EU), since the epidemiology of antimicrobial resistance varies markedly between these regions.

## Materials and methods

### Organism collection

Each participating centre was asked to collect 100 (W-EU and E-EU) or 120 (USA) consecutive bacterial isolates each year from lower respiratory tract specimens determined to be significant by local criteria as the reported probable cause of pneumonia. Medical records were not available to make clinical inferences about the infection source for patients hospitalized with pneumonia (e.g. community-acquired or hospital-acquired); thus, this category includes patients hospitalized for any reason who were diagnosed with pneumonia while in the hospital. Qualified sputum samples and isolates from invasive sampling (transtracheal aspiration, bronchoalveolar lavage, protected brush samples, etc.) were accepted. The participating laboratory identified isolates and then the reference monitoring laboratory (JMI Laboratories) confirmed bacterial identifications by standard algorithms and/or by MALDI-TOF MS (Bruker Daltonics, Bremen, Germany).

A total of 28 918 bacterial isolates were collected (one per patient) in 2016–19 from 121 medical centres located in W-EU (*n =* 7966; 25 centres from 10 countries), E-EU (*n =* 3182; 14 centres from 11 countries) and the USA (*n =* 17 770; 82 centres). The W-EU countries surveyed were Belgium, France, Germany, Ireland, Italy, Portugal, Spain, Sweden, Switzerland and the United Kingdom. The E-EU countries included Belarus, Croatia, the Czech Republic, Greece, Hungary, Poland, Romania, Russia, Slovakia, Slovenia and Turkey.

Carbapenem-resistant Enterobacterales (CRE) was defined as any isolate displaying MIC values of >2 mg/L for imipenem and/or meropenem. Imipenem was not applied for *Proteus mirabilis* or indole-positive Proteeae due to their intrinsically elevated MIC values.

### Susceptibility methods

Organisms were tested for susceptibility by reference broth microdilution methods in a central laboratory according to the current CLSI documents.^7^ Validated MIC panels (frozen-form) were manufactured at JMI Laboratories. Susceptibility percentages were based on EUCAST breakpoint criteria, where available.^8^ CLSI or US FDA breakpoints were applied when EUCAST breakpoints were not available.^9^

### Screening for β-lactamases

CRE isolates were tested for β-lactamase-encoding genes using next-generation sequencing (NGS). Total genomic DNA was extracted using the fully automated Thermo Scientific™ KingFisher™ Flex Magnetic Particle Processor (Cleveland, OH, USA). To perform NGS, DNA extracts were quantified using the Qubit™ High Sensitivity DS-DNA assay (Invitrogen, Thermo Fisher Inc.) and normalized to 0.2 ng/μL. A total of 1 ng high-quality genomic DNA was used as input material for library construction using the Nextera XT™ DNA library preparation kit (Illumina, San Diego, CA, USA). Libraries were normalized using the bead-based normalization procedure (Illumina) and sequenced on MiSeq. The generated FASTQ files were assembled using SPAdes Assembler and subjected to a proprietary software (JMI Laboratories) for screening of β-lactamase genes.^10^

## Results

Gram-negative bacilli (GNB) represented 76.3%, 88.6% and 69.1% of organisms isolated from respiratory samples of patients hospitalized with probable pneumonia in W-EU, E-EU and the USA, respectively, and non-fermentative (NF) GNB accounted for 26.9%, 51.8% and 34.6% of organisms in W-EU, E-EU and the USA, respectively. Overall, *Pseudomonas aeruginosa* (*n =* 6828), *Staphylococcus aureus* (*n =* 6732) and *Klebsiella pneumoniae* (*n* = 2780) were the most prevalent organisms, but frequency varied among regions (Figure S1, available as Supplementary data at *JAC-AMR* Online). *P. aeruginosa* ranked first in W-EU (20.6%) and E-EU (27.2%) and second in the USA (24.3%); whereas *S. aureus* was most common in the USA (27.3%), second in W-EU (20.1%) and fourth in E-EU (9.1%). *K. pneumoniae* ranked second in E-EU (19.3%), third in the USA (8.1%) and fourth in W-EU (9.2%). Among other NF-GNB (besides *P. aeruginosa*), *Acinetobacter baumannii* ranked third in E-EU and accounted for 19.0% of the organisms from that region, and *Stenotrophomonas maltophilia* was among the top eight in all three regions, with frequencies of 4.7% in the USA, 3.9% in E-EU and 3.2% in W-EU. The frequencies of the top 12 organisms isolated from respiratory samples of patients hospitalized with probable pneumonia stratified by geographic region are shown in Figure S1.

During the investigation period (2016–19), the yearly frequency of *P. aeruginosa* decreased from 22.6% to 20.7% in W-EU and from 28.2% to 25.8% in E-EU. Likewise, *A. baumannii* decreased from 2.5% to 1.5% in W-EU and from 21.3% to 18.4% in E-EU. However, *S. maltophilia* decreased in W-EU from 4.5% to 2.0% but increased in E-EU from 3.5% to 5.6% (data not shown). *S. aureus* and *K. pneumoniae* increased in E-EU and remained stable in W-EU. In the USA, the frequency of *S. aureus* decreased from 29.4% in 2016 to 23.9% in 2019; whereas the frequency of *P. aeruginosa* increased from 23.4% in to 2016 to 25.2% in 2019. Thus, in 2019, *P. aeruginosa* ranked first (25.2%) and *S. aureus* second (23.9%) in the USA (data not shown).

Colistin was active against 99.3% (E-EU) to 99.7% (USA) of *P. aeruginosa* isolates based on current EUCAST breakpoints. After colistin, ceftazidime/avibactam and ceftolozane/tazobactam were the most active agents against *P. aeruginosa* in all three geographic regions and exhibited similar coverage against these organisms. These two β-lactamase inhibitor combinations were active against 93.9% to 96.8% of *P. aeruginosa* isolates from W-EU and the USA, but susceptibility rates were markedly lower among E-EU isolates (80.8%–82.9%; Table 1). *P. aeruginosa* susceptibility rates to most antimicrobial agents were similar in W-EU and the USA and markedly lower in E-EU. *P. aeruginosa* susceptibility to piperacillin/tazobactam (defined as an MIC value of ≤16 mg/L) varied from 75.4% in W-EU and 76.1% in the USA to 57.4% in E-EU. Moreover, the occurrence of piperacillin/tazobactam-non-susceptible *P. aeruginosa* (MIC >16 mg/L) decreased in W-EU from 28.8% in 2016 to 22.2% in 2019 and in the USA from 23.8% in 2016 to 21.1% in 2019 but increased in E-EU from 39.6% in 2016 to 47.2% in 2019 (Table 2). Similar trends were observed with frequency of *P. aeruginosa* isolates non-susceptible to meropenem (MIC >2 mg/L) and ceftazidime (MIC >8 mg/L), i.e. there was a decrease in W-EU and the USA and an increase in E-EU during the study period (data not shown).

**Table 1:** Antimicrobial susceptibility of the main organisms isolated from patients hospitalized with pneumonia from western Europe (W-EU), eastern Europe (E-EU) and the USA

Organism/Antimicrobial agent	Percentage susceptible (no. of isolates)^a^			
	W-EU	E-EU	USA	
*P. aeruginosa*	(1643)	(864)	(4321)	
ceftazidime^b^	79.2	63.2	81.0	
ceftazidime/avibactam	96.5	82.9	96.4	
ceftolozane/tazobactam	93.9	80.8	96.8	
piperacillin/tazobactam^b^	75.4	57.4	76.1	
meropenem	76.9	48.3	74.8	
levofloxacin^b^	68.0	40.7	60.9	
tobramycin	87.0	65.4	88.7	
colistin	99.5	99.3	99.7	
*S. aureus*	(1598)	(289)	(4845)	
oxacillin	78.6	71.3	56.3	
ceftaroline	97.4	94.8	96.4	
clindamycin	93.9	87.8	80.4	
doxycycline	96.8	98.6	95.9	
levofloxacin^b^	77.7	83.4	61.1	
trimethoprim/sulfamethoxazole^b^	98.3	99.3	98.2	
*K. pneumoniae*	(733)	(615)	(1432)	
ceftriaxone	70.1	34.5	80.7	
ceftazidime/avibactam	99.2	92.0	100.0	
ceftolozane/tazobactam	87.3	56.7	92.3	
piperacillin/tazobactam	71.4	38.7	78.3	
meropenem	91.3	72.5	95.3	
levofloxacin	71.2	39.2	82.6	
gentamicin	80.8	55.3	89.7	
*E. coli*	(1015)	(195)	(1132)	
ceftriaxone	79.2	62.6	71.4	
ceftazidime/avibactam	99.9	99.5	100.0	
ceftolozane/tazobactam	98.8	98.5	95.8	
piperacillin/tazobactam	84.2	85.6	85.1	
meropenem	99.6	99.5	99.5	
levofloxacin	71.2	55.9	55.1	
gentamicin	89.0	79.0	84.2	
*E. cloacae*	(446)	(100)	(698)	
ceftazidime	64.7	61.0	68.0	
cefepime	82.1	69.0	83.1	
ceftazidime/avibactam	99.1	99.0	100.0	
ceftolozane/tazobactam	83.6	83.4	78.1	
piperacillin/tazobactam	71.4	73.0	73.4	
meropenem	99.8	98.0	98.0	
levofloxacin	89.6	82.0	92.5	
gentamicin	91.3	78.0	94.3	
*S. marcescens*	(339)	(73)	(772)	
ceftriaxone	89.4	85.1	83.1	
ceftazidime/avibactam	100.0	100.0	99.7	
ceftolozane/tazobactam	97.3	97.3	97.2	
piperacillin/tazobactam	92.1	94.6	87.8	
meropenem	100.0	100.0	98.4	
levofloxacin	90.0	91.9	89.1	
gentamicin	98.5	90.5	96.5	
*A. baumannii*	(153)	(604)	(493)	
ceftazidime^b^	45.8	6.8	56.2	
piperacillin/tazobactam^b^	39.7	6.0	49.3	
meropenem	45.8	10.4	58.8	
levofloxacin	42.5	6.0	53.1	
amikacin	55.6	13.9	73.0	
tobramycin	56.2	33.4	75.7	
colistin	98.0	82.3	91.3	
*S. maltophilia*	(252)	(125)	(836)	
ceftazidime^b^	14.3	16.0	18.1	
minocycline^b^	100.0	100.0	99.2	
levofloxacin^b^	83.7	84.0	74.5	
trimethoprim/sulfamethoxazole	96.4	94.3	94.0	

a Criteria as published by EUCAST (2020), unless noted.

b Based on CLSI criteria.

**Table 2.** Frequency of key resistance phenotypes stratified by year

Resistance phenotype/Geographic region	Frequency (%) of resistance phenotype			
	2016	2017	2018	2019
Piperacillin/tazobactam non-susceptible *P. aeruginosa*				
W-EU	28.8	24.1	22.9	22.2
E-EU	39.6	40.2	44.5	47.2
USA	23.8	25.2	24.8	21.1
MRSA				
W-EU	29.2	20.8	19.7	16.1
E-EU	32.8	23.5	17.5	38.6
USA	44.8	44.5	42.9	40.1
Meropenem non-susceptible *K. pneumoniae*				
W-EU	14.6	5.2	9.4	5.6
E-EU	20.1	29.3	25.9	33.5
USA	6.7	3.5	4.2	3.0
Meropenem non-susceptible *A. baumannii*				
W-EU	65.3	74.2	45.2	29.0
E-EU	93.1	86.3	84.2	93.0
USA	45.4	46.3	35.8	29.6
Carbapenem-resistant Enterobacterales				
W-EU	4.0	1.8	2.0	1.4
E-EU	10.5	15.8	17.2	23.6
USA	3.0	2.5	1.8	1.7

W-EU, western Europe; E-EU, eastern Europe.

Overall MRSA rates were 21.4% in W-EU, 28.7% in E-EU and 43.7% in the USA (Table 1). MRSA rates decreased from 29.2% in 2016 to 16.1% in 2019 in W-EU and from 44.8% in 2016 to 40.1% in 2019 (*P <* 0.05) in the USA. In E-EU, MRSA rates were higher in 2016 (32.8%) and 2019 (38.6%) than 2017 (23.5%) and 2018 (17.5%; Table 2). It is important to note that dalbavancin (MIC_50_ and MIC_90_ 0.03 mg/L), linezolid (MIC_50/90_ 1/2 mg/L), oritavancin (MIC_50_ and MIC_90_ 0.03 mg/L), teicoplanin (MIC_50_ and MIC_90_ ≤0.5 mg/L), telavancin (MIC_50/90_ 0.03/0.06 mg/L), tigecycline (MIC_50/90_ 0.06/0.12 mg/L) and vancomycin (MIC_50_ and MIC_90_ 1 mg/L) were active against ≥99.9% of *S. aureus* isolates overall (data not shown). Moreover, trimethoprim/sulfamethoxazole and ceftaroline exhibited good activity against *S. aureus* in all three regions, with susceptibility rates of 98.2%–99.3% for trimethoprim/sulfamethoxazole and 94.8%–97.4% for ceftaroline (Table 1).

Among *K. pneumoniae*, susceptibility rates were significantly lower in E-EU compared with W-EU and the USA. *K. pneumoniae* susceptibility rates for ceftriaxone and meropenem were 70.1% and 91.3% in W-EU, 34.5% and 72.5% in E-EU and 80.7% and 95.3% in the USA, respectively (Table 1). Notably, the susceptibility of *K. pneumoniae* to meropenem increased during the study period from 85.4% to 94.4% in W-EU and from 93.3% to 97.0% in the USA but decreased from 79.9% to 66.5% in E-EU (Table 2).

*Escherichia coli* represented 12.7%, 6.1% and 6.4% of isolates from W-EU, E-EU and the USA, respectively (Figure S1), and exhibited susceptibility rates for ceftriaxone and levofloxacin of 79.2% and 71.2% in W-EU, 62.6% and 55.9% in E-EU and 71.4% and 55.1% in the USA, respectively (Table 1). Meropenem and ceftazidime/avibactam were highly active against *E. coli* (≥99.5% susceptibility) from all geographic regions (Table 1. Meropenem (98.0%–99.8% susceptibility) and ceftazidime/avibactam (99.0%–100.0% susceptibility) were also highly active against *Enterobacter cloacae* species complex isolates from all three regions, whereas all other compounds tested exhibited limited activity against these organisms, especially against isolates from E-EU (Table 1). Many compounds demonstrated good activity (>90.0% susceptibility) against *Serratia marcescens* (Table 1).

CRE rates varied widely among regions and over time. In E-EU, CRE increased continuously from 10.5% in 2016 to 23.6% in 2019 (Table 2). In contrast, CRE rates decreased significantly in W-EU from 4.0% in 2016 to 1.4% in 2019. CRE rates also decreased in the USA from 3.0% in 2016 to 1.7% in 2019 (Table 2). *K. pneumoniae* represented 82.9%, 93.4% and 55.7% of CRE isolates from W-EU, E-EU and the USA, respectively.

Results of β-lactamase screening on CRE isolates indicated that although the main mechanism of carbapenem resistance is the production of a carbapenemase in all three geographic regions analysed, the type of carbapenemase varied substantially (Table 3). The KPC class predominated in W-EU (68.3% of CRE) and the USA (84.7% of CRE), while OXA type (mainly OXA-48) and MBLs (mainly NDM-1) were the most common carbapenemases found in E-EU. OXA carbapenemases and MBLs were detected in 45.8% and 28.1% of CRE isolates from E-EU, respectively, and comprised 88.2% of all carbapenemases found in CRE isolates from this region (Table 3).

**Table 3.** Summary of carbapenemases observed among carbapenem-resistant Enterobacterales (CRE) stratified by geographic region

β-Lactamase class	No. of isolates (% within the region)		
	W-EU	E-EU	USA
KPC	56 (68.3)	19 (9.9)	111 (84.7)
KPC-2	4 (4.9)	12 (6.3)	40 (30.5)
KPC-3	52 (63.4)	6 (3.1)	69 (52.7)
other KPCs^a^	0 (0.0)	1 (0.5)	2 (1.5)
OXA	11 (13.4)	88 (45.8)	1 (0.8)
OXA-48	10 (12.2)	73 (38.0)^f^	1 (0.8)
other OXAs^b^	1 (1.2)	15 (7.5)^f^	0 (0.0)
SME^c^	0 (0.0)	0 (0.0)	3 (2.3)
MBL	10 (12.2)	54 (28.1)	1 (0.8)
NDM-1	6 (7.3)	44 (22.9)^f^	1 (0.8)
VIM^d^	4 (4.9)	10 (5.2)	0 (0.0)
Negative^e^	3 (3.7)	31 (16.1)	15 (11.5)
Not tested	2 (2.4)	4 (2.1)	0 (0.0)
Total	82 (100.0)	192 (100.0)	131 (100.0)

W-EU, western Europe; E-EU, eastern Europe.

a Includes KPC-12 (E-EU), KPC-6-like (USA) and KPC-56 (USA).

b Includes OXA-181 in E-EU and W-EU (2 isolates), OXA-232 (9 isolates) and OXA-244 (5 isolates) in E-EU.

c Includes SME-1 (1 isolate) and SME-4 (2 isolates).

d Includes VIM-1 (4 isolates in W-EU and 6 isolates in E-EU), VIM-19 (3 isolates in E-EU) and VIM-4 (1 isolate in E-EU).

e No carbapenemase genes were identified by WGS.

f Three isolates had an NDM-1 and an OXA-48 and one isolate had an NDM-1 and an OXA-181.

*A. baumannii* represented 1.9%, 19.0% and 2.8% of isolates from W-EU, E-EU and the USA, respectively (Figure S1), and exhibited high resistance rates to most agents tested (Table 2). The only compounds active against >50.0% of isolates overall for all regions combined were colistin (MIC_50/90_ 0.5/4 mg/L; 87.8% susceptible) and tobramycin (MIC_50/90_ 4/>8 mg/L; 52.9% susceptible). Overall susceptibility rates to meropenem were 45.8%, 10.4% and 58.8% in W-EU, E-EU and the USA, respectively (Table 1), but those rates varied markedly over time in W-EU and the USA. Meropenem susceptibility increased from 34.7% in 2016 to 71.0% in 2019 in W-EU and from 54.6% to 70.4% during the same time period in the USA (Table 2).

*S. maltophilia* was the second most common NF-GNB in W-EU (3.2% of total) and in the USA (4.7%), and it represented 3.9% of organisms from E-EU (Figure S1). The most active compounds against *S. maltophilia* were minocycline (99.2%–100.0% susceptibility per CLSI) and trimethoprim/sulfamethoxazole (94.3%–96.5% susceptibility per EUCAST; Table 1).

## Discussion

The SENTRY programme monitors the frequency of organisms and antimicrobial resistance of bacteria from respiratory samples of patients hospitalized with probable pneumonia worldwide since 1997 and this investigation updates our previous evaluation of the first 20 years of SENTRY (1997–2016).^1^^,^^6^ In our previous investigation, we showed that the frequency and antimicrobial susceptibility of organisms isolated from respiratory samples of patients hospitalized with probable pneumonia varied markedly by geographic region and over time. Results from other SENTRY programme investigations as well as from other European surveillance programmes also have shown a marked regional variation within Europe. Many reports have revealed a north-to-south and west-to-east gradient of resistance rates for many organism–antimicrobial combinations, with lower resistance rates being reported by countries in the north and west of Europe and higher resistance rates being reported by countries in the south and east.^11^^,^^12^

Our previous investigation also showed an increasing frequency of Gram-negative organisms recovered from respiratory samples of patients with probable pneumonia in Europe during 1997–2016, accompanied by an increasing resistance to key antimicrobial agents among these organisms.^6^ In contrast, the results of this investigation indicated that the frequencies of major Gram-negative organisms decreased or remained stable in both W-EU and E-EU in 2016–19, except for *E. coli* in W-EU and *K. pneumoniae* in E-EU. Moreover, resistance rates for key antimicrobial agents generally decreased among Gram-negative organisms from W-EU in 2016–19 but increased markedly among key Gram-negative organisms in the E-EU region, especially *P. aeruginosa* and *K. pneumoniae*. Similarly, MRSA rates decreased in W-EU and increased in E-EU during the period of this investigation (Table 2). Thus, our results emphasize the diversity and instability of the epidemiology of antimicrobial resistance in Europe.

Regarding the USA, our previous investigation showed a persistent decrease in the frequency of *S. aureus* and MRSA rates during 2005–16,^6^ and these results show that this trend continued in 2016–19 (Table 2). It is important to note, however, that MRSA rates and trends vary markedly by region.^13^ The increasing frequency of *P. aeruginosa* and *S. maltophilia* from 2005–16 persisted during 2016–19. Moreover, trimethoprim/sulfamethoxazole resistance among *S. maltophilia* continued to increase, whereas resistance to piperacillin/tazobactam among *P. aeruginosa* and resistance to carbapenems among Enterobacterales (CRE rates) continued to decrease in 2016–19.^6^

The fact that the criteria used to categorize a bacterial isolate as clinically significant were not defined in the study protocol and were based on local algorithms is a limitation of this study, since these criteria can vary among participating medical centres. Also, due to the lack of clinical information available, we cannot exclude the possibility that some organisms were colonizers. It is also important to note that a limited number of isolates and/or medical centres were surveyed in some European countries; thus, the results presented here may not represent the overall picture from those countries. Another study limitation is that some medical centres did not participate in the programme during the entire investigation period. Also, the criteria used to define CRE allowed isolates to be included in this group that could be considered intermediate to imipenem and/or meropenem per EUCAST criteria. These limitations should be considered when interpreting the results and conclusions, but it is improbable that they have introduced important bias to the study.

In summary, our results clearly indicate that the frequency and antimicrobial resistance of organisms isolated from respiratory samples of patients with probable pneumonia varied markedly by geographic region and over time, emphasizing the importance of continued surveillance through large multicentre programmes. Although resistance rates and microbial epidemiology may vary substantially by geographic region and even from hospital to hospital, results from a large, well-monitored surveillance network, such as those presented here, can provide useful information by detecting signs of emerging pathogen populations and/or resistance patterns as well as valuable data on trends of antimicrobial resistance phenotypes and genotypes.

## Supplementary Material

dlab117_Supplementary_Data
