# Supplementary material for: Frequency of occurrence and antimicrobial susceptibility of bacteria isolated from respiratory samples of patients hospitalized with pneumonia in Western Europe, Eastern Europe and the USA: results from the SENTRY Antimicrobial Surveillance Program (2016–19)
Source: JAC Antimicrob Resist. 2021 Sep 2;3(3):dlab117. doi: 10.1093/jacamr/dlab117 (PMC8522161; doi:10.1093/jacamr/dlab117)

**Supplementary data**

**Figure S1**. Frequency of occurrence of organisms isolated from patients hospitalized with pneumonia stratified by geographic region (2016-2019)


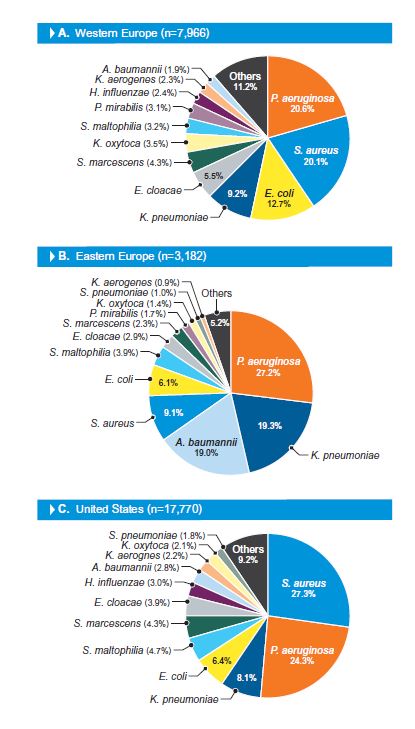

Supplement: dlab117_Supplementary_Data [file dlab117_supplementary_data.docx]
